# Solid State Fermentation of Olive Leaves as a Promising Technology to Obtain Hydroxytyrosol and Elenolic Acid Derivatives Enriched Extracts

**DOI:** 10.3390/antiox11091693

**Published:** 2022-08-29

**Authors:** Anna Starzyńska-Janiszewska, Carmen Fernández-Fernández, Beatriz Martín-García, Vito Verardo, Ana María Gómez-Caravaca

**Affiliations:** 1 Department of Biotechnology and General Technology of Food, Faculty of Food Technology, University of Agriculture in Krakow, Balicka 122, 30-149 Krakow, Poland; 2 Department of Analytical Chemistry, Faculty of Sciences, University of Granada, Av. de Fuentenueva s/n, 18071 Granada, Spain; 3 Department of Nutrition and Food Science, University of Granada, Campus of Cartuja, 18071 Granada, Spain; 4 Institute of Nutrition and Food Technology ‘José Mataix’, Biomedical Research Centre, University of Granada, Avda del Conocimiento s/n, 18100 Armilla, Spain

**Keywords:** olive leaves, fermentation, phenolic compounds, hydroxytyrosol, antioxidant activity

## Abstract

Extraction of valuable bioactive compounds from olive leaves is a hot topic and the use of sustainable and green technologies is mandatory in terms of circular economy. In this way, the use of fermentation technologies showed very interesting results in terms of phenolic compound recovery. Because of that in this work the use of solid state fermentations, as valuable tool to improve the phenolic extraction has been checked. *Aspergillus oryzae* (in mycelium and spore form), *Aspergillus awamori* and *Aspergillus niger* were used as fermentation microrganisms. Phenolic compounds were determined by HPLC-ESI-TOF-MS and, to our knowledge, new compounds have been tentatively identified in olive leaves. Fermentation using mycelium of *Aspergillus awamori*, *Aspergillus niger* and *Aspergillus oryzae* were effective to increase both hydroxytyrosol and elenolic acid derivatives whereas the use of spores of *Aspergillus oryzae* caused a loss of hydroxytyrosoyl derivatives, contrary the content of elenolic derivatives are comparable with the other fermentation treatments and higher than control. The proposed fermentation processes using the mycelium of *Aspergillus awamori*, *Aspergillus niger* and *Aspergillus oryzae* lead to an increase the hydroxytyrosyl and elenolic acid derivatives and could be used at industrial scale to obtain enriched extracts.

## 1. Introduction

Olive (*Olea europaea* L.) is an evergreen tree found throughout the world, especially in the Mediterranean region, and cultivated for the production of oil and table olives [1,2]. Despite the important role played by olive industry within economy and society, its rapid growth and extensive activity generate large amounts of residues and by-products that can have a negative impact on environment, when burnt or grinded and scattered on the field, due to their high organic content and phytotoxicity [3,4]. Thus, valorization of by-products as a source of value-added products seems a potential alternative to increase the profitability of the olive sector. 

One of the most important by-products from both olive tree cultivation and olive processing industry are olive leaves, generated in large quantities every year (10–30 kg per tree) after being separated from olives before processing (5% of weight of olives) and during the pruning of olive trees (25% of weight of olives) [5,6]. These by-products have always been used as animal feed and in folk medicine. However, they have the potential to be used as a valuable raw material in different fields, especially in the pharmaceutical and food industries [7,8]. In fact, olive leaves are rich in a wide variety of phenolic compounds, supporting their use in medicine, pharmaceutics, cosmetics, food shelf-life improvement and functional foods development [9,10,11,12]. Therefore, olive leaves not only are a by-product produced in large amounts, but also an excellent source of bioactive compounds, highlighting the potential valorization of this unexploited resource. Various studies have evaluated olive leaf extracts properties in relation to their phenolic content, describing better results in terms of antioxidant capacity when mixed than when compounds are tested separately due to the synergic effect between phenols [13,14]. In this sense, utilization of olive leaf extracts rather than an isolated compound would contribute to a better by-product valorization. 

Phenolic compounds are generally extracted from natural sources through solid-liquid extractions using organic solvents; but there are other existing techniques proposed to obtain them, including supercritical fluids, high pressure processes, microwave-assisted extraction, and ultrasound-assisted extraction [15]. Recently, fermentation has also gained attention since it is able to provide high quality and high activity extracts which are easily recovered, enhancing the profitability of this process [16]. Nowadays, there are two main strategies to carry out fermentation process, depending on the type of substrate: submerged fermentation (SmF) and solid-state fermentations (SSF). In the first one, a liquid substrate (or water added to solid substrate) is used for microorganisms cultivation, while in SSF fermenting microorganisms grow on a solid substrate under controlled conditions, in the absence of free water (or with minimal amount of it) [17]. SSF shows several advantages over SmF, such as similarity to the natural habitat of microorganisms, a high yield, obtaining a final product with high activity, low water and energy consumptions, greater resistance to contamination, and being more environmentally friendly and cheaper as it can use agro-industrial by-products as substrates for fermentation, reducing the waste generated [17,18,19]. Consequently, there is an increasing interest towards the application of SSF for the recovery of valuable bioactive compounds from agro-industrial by-products, as this process proves to be more efficient than SmF [20]. 

Although SSF can be performed with different microorganisms, filamentous fungi are the most commonly used microorganisms in this type of fermentation, because of their ability to grow under low moisture content conditions and their potential to produce bioactive compounds by SSF as a result of enzymatic activity [19,21]. Indeed, filamentous fungi of genera Aspergillus have being widely used for studying the influence of fermentation on phenolic profiles from different agro-industrial by-products and enhancing their antioxidant potential [22,23,24,25,26,27,28]. There is limited information regarding the use of olive leaves as substrate for SSF; just about the effects of *A. niger* on nutrient and mineral composition, phenolic compounds, and condensed tannin of olive leaves, in relation to animal feed [29,30]. Therefore, the potential of this by-product together with SSF remains unknown. 

In this context, the aim of the present study was to evaluate the influence of SSF on phenolic content and thereby on antioxidant activity of olive leaves samples, concerning the potential of this fermented by-product as an excellent source of bioactive compounds for pharmaceutical and food applications. For that purpose, fermentation process was carried out with three different strains of Aspergillus (*A. awamori*, *A. niger* and *A. oryzae*) at different fermentation periods comparing the phenolic profiles obtained.

## 2. Materials and Methods

### 2.1. Sample and Microorganism Strains

Olive leaves from ‘Picual’ cultivar were obtained from “IFAPA, Centro Alameda del Obispo” in Córdoba, Spain (37°51′36.5″ N, 4°47′53.7″ W). Olive leaves were air dried at room temperature. Then, leaves were ground with an IKA A 10 Basic Mill (Retsch GmbH, Haan, Germany) and the obtained powder was stored at −20°C until the extraction.

Fungal strains tested in the study were: *Rhizpus oligosporus* ATCC 64063, *Neurospora intermedia* CBS 131.92, *Aspergillus oryzae* CBS 673.92, *Aspergillus awamori* KKP 40 (IBPRS-Collection of Industrial Microbial Cultures (KKP), Institute of Agricultural and Food Biotechnology, Warsaw, Poland) and *Aspergillus niger* 377-4 (Fruit and Vegetable Industry Factory Pektowin, Jasło, Poland). The strains were grown on potato extract agar (PDA) slants for 12 days at 24 °C.

### 2.2. Fermentation Process

**Figure 1:**
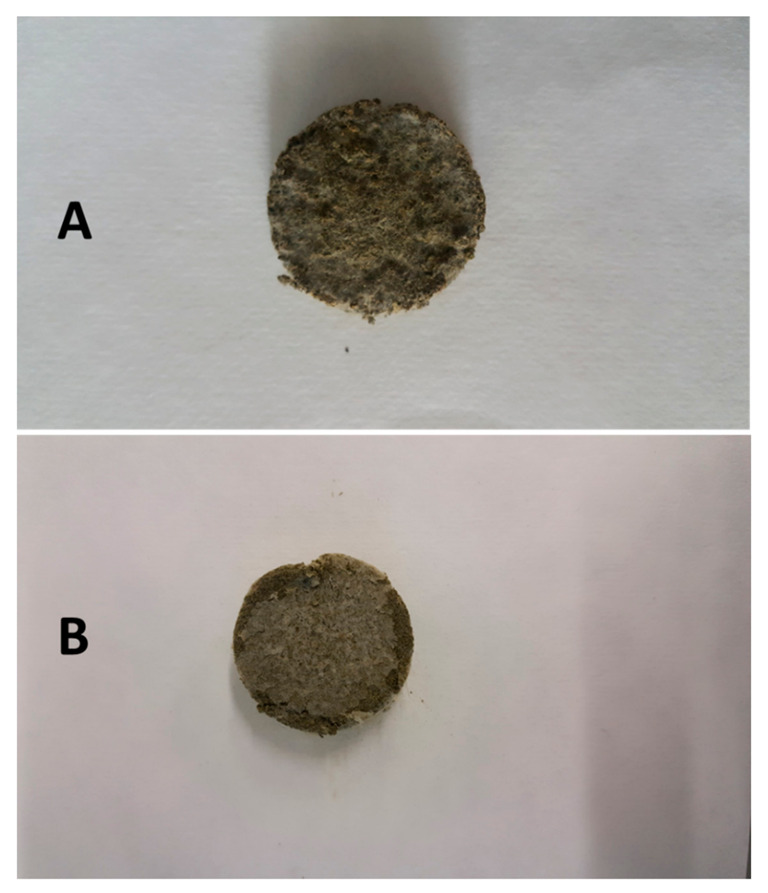
*A. oryzae* 5 days inoculated with mycelium (**A**) and *A. oryzae* 8 days inoculated with spores (**B**).

Dry milled olive leaves were hydrated to 65% moisture content with distilled water, supplemented with yeast extract (1 g/L), NaHPO_4_ (1 g/L), MgSO_4_ (0.5 g/L) and KCl (0.5 g/L) to encourage fungal growth. Then, the material was autoclaved (121 °C, 20 min). Cooled material was inoculated with spore suspension (10^6^ spores per 25 g of raw substrate) or mycelium fragments (3 loops per 25 g of raw substrate) of *R. oligosporus*, *N. intermedia* or *A. oryzae* strain. Inoculated substrate was transferred into Petri dishes (3 cm diameter, 3 replicates for each variant) and incubated at 30 °C and 60% air humidity. Among the strains tested, only *A. oryzae* was capable of growing on olive leaves. It was previously reported that olive leaf extract showed inhibitory effect on some fungal strains, concerning spore germination and/or mycelium growth [31]. The substrate was fully overgrown by mycelium after 5 days (AO5-m) (Figure 1A) and 8 (AO8-s) days (Figure 1B), respectively, when mycelium and spores were used as inoculating material. 

As mycelium proved to be more effective than spores, the former procedure was also applied for *A. awamori* (Aaw) and *A. niger* (Anig) strains, and the inoculated material was incubated for 5 days. The fermentation was stopped by steaming (10 min), and then material was lyophilized.

### 2.3. Extraction of Phenolic Compounds from Fermented Olive Leaves 

The extraction was performed according to the methodology described by Martín-García et al. [32] Briefly, 0.25 g of fermented material were extracted by sonication via sonotrode with 100 mL of an ethanol/water solution (55:45, *v*/*v*) for 8 min. The supernatant was evaporated and reconstituted with 3 mL of MeOH/H_2_O (50/50 *v/v*). The final extracts were filtered through 0.22 μm nylon syringe filters and stored at −18 °C until the analyses.

### 2.4. Determination of Phenolic Compounds in Fermented Olive Leaves by HPLC-ESI-TOF-MS Analysis

The determination of the phenolic composition of fermented olive leaves was conducted slightly modifying the method previously described by Talhaoui et al. [33]. The equipment consists of a UPLC system ACQUITY (Waters Corporation, Milford, MA, USA) coupled to a time-of-flight analyzer (TOF) (Waters Corporation, Milford, MA, USA). Phenolic compounds were separated with a Poroshell 120 EC-C18 analytical column (4.6 × 100 mm, 2.7 mm) from Agilent Technologies, maintaining the column temperature at 25 °C, a flow rate set at 0.8 mL min^−1^ and an injection volume of 2.5 µL. The mobile phases were water with 1% acetic acid (phase A) and acetonitrile (phase B), changing the solvent gradient as follows: 0 min, 5% B; 4 min, 9% B; 7 min, 12% B; 8 min, 15% B; 9 min, 16% B; 14 min, 20% B; 15 min, 22% B; 18 min, 28% B; 19 min, 30% B; 20 min, 31% B; 24 min, 40% B; 28 min, 100% B; 31 min, 100% B; 33 min, 5% B. Mass spectrometer was equipped with an interface with electrospray ionization (ESI) source operating in negative mode. The operational conditions were: capillary voltage, 2300 kV; source temperature, 100 °C; cone gas flow, 40 L/h; desolvatation temperature, 500 °C; desolvatation gas flow, 11,000 L/h; and scan range, *m/z* 50–1500. The software MassLynx 4.1 (Waters Corporation, Milford, MA, USA) was used to process the data acquired. 

The quantification of phenolic compounds in the fermented extracts was performed by using five standards (hydroxytyrosol, apigenin-7-glucoside, rutin, luteolin and oleuropein). Their calibration curves were prepared at eight concentration levels from the limit of quantification (LOQ) to 250 ppm (Table S1).

### 2.5. Antioxidant Capacity Assays

Antioxidant capacity has been evaluated using three different assays called DPPH, ABTS and FRAP according to our previous study [32]. All the spectrophotometric assays performed using on aUV-1601spectrophotometer from Shimadzu (Duisburg, Germany). Results were expressed in Trolox equivalents using its calibration curve.

To evaluate the DPPH radical scavenging, 100 µL of each extract was added to 2.9 mL of 0.1 M DPPH solution in 80/20 methanol/water (*v*/*v*). A decrease in absorbance was evaluated at 517 nm in the 0–30 min range (at 25 °C).

The radical-scavenging capability of phenolic was tested using the ABTS•+ radical cation assay (2,2′-azinobis(3-ethylbenzothiazoline)-6-sulfonic acid, diammonium salt; a stock solution of ABTS 7 mM was prepared and diluted with phosphate buffer 5 mM at pH = 7.4 obtaining an absorbance of 0.7 ± 0.02. The extracts were added to the solution and the absorbance was monitored for 10 min at 734 nm. 

The ability of phenolic compounds to reduce the ferric ions by a single electron-transfer mechanism was evaluated by the ferric reducing antioxidant power (FRAP) assay; freshly prepared FRAP reagent (containing TPTZ, FeCl3 and acetate buffer) was warmed at 37 °C and added of the phenolic extracts. The absorbance values were measured at 595 nm after 30 min.

### 2.6. Statistical Analysis

The results reported in this work are the averages of three repetitions (n = 3). Fisher test was used to establish significant differences (one-way ANOVA) (at the *p* < 0.05 level) that were calculated using Statistica 6.0 software (2001, StatSoft, Tulsa, OK, USA). PLS-DA, VIP and Hierarchical Clustering results presented as heatmaps have been obtained with MetaboAnalyst 4.0 software.

## 3. Results and Discussion

### 3.1. Identification of Phenolic Compounds by HPLC-ESI-TOF-MS

A total of 96 compounds have been identified and 74 phenolic compounds have been quantified in the samples analyzed by HPLC-ESI-TOF-MS. They were identified according to their *m/z*, molecular formula, in source fragments, error and Fit Conf % and by comparing them with literature, the co-elution with commercial standards (when possible) and with several databases (Pubchem, KEGG COMPOUND Database). 

Among them, 5 compounds belonged to simple phenols, 6 to elenolic acid derivatives, 18 to flavonoids, 40 to secoiridoids, 1 to iridoids and 4 to other phenolic compounds (Table S2).

Main of them was previously described in olive leaves and olive leaf extracts. However, to our knowledge few compounds were tentatively described for the first time in olive leaves. The compound 10 with molecular formula C_10_H_16_O_5_ and *m/z* 215 was tentatively assigned to elenolic acid derivative according to Abbattista et al. [34] that identified them in olive pomaces. Compounds 15 and 16 showed an *m/z* 639 and a molecular formula C_29_H_36_O_16_; Cardinali and co-workers [35] identified this compound in olive mill wastewaters assigning it to β-Hydroxyverbascoside. Compounds 18 and 20, which were tentatively identified only in fermented samples, showed a molecular ion at 243 *m/z* and molecular formula C_11_H_16_O_6_; several authors [36] described them in plasma and olive pomaces assigning it to hydrogenated-elenolic acid. Compound 31 with molecular ion at *m/z* 551 and molecular formula C_26_H_32_O_13_ was tentatively identified as 6-(3-Hydroxy-3-methylbutyl) taxifolin 7-O-β-D-glucoside and it was found only in some fermented samples. Compound 39 reported a molecular formula of C_17_H_22_O_7_ and a molecular ion at 337 *m/z*, according to Ventura et al. [37] it was tentatively assigned to monohydrated oleacein and it was identified only in fermented samples. Compound 40 with *m/z* 323 was tentatively identified as hydroxytyrosol derivative according to Garcia-Aloy [38]. Compound 52 with 321 *m/z* and molecular formula C_17_H_22_O_6_ was tentatively identified as lactone ester with hydroxytyrosol according to the data reported by Garcia-Aloy and co-workers [38]. Compounds 40 and 52 were identified in all fermented samples but not in the control.

Finally, compounds 81 with *m/z* 601 and molecular formula C_27_H_38_O_15_ was tentatively identified as frameroside or 2″-epi-frameroside; Sidda and co-workers [39] identified them in ash leaves from *Oleaceae* family.

### 3.2. Concentration of Phenolic Compounds by HPLC-ESI-TOF-MS

The control sample showed a total phenolic content of 14.64 mg/g d.w. (Table S3), a lower content than the presented in other studies about ‘Picual’ olive leaves (32–34), maybe due to the high temperatures reached during autoclaving and also because of differences in agronomic and environmental conditions. Total phenolic content showed a significant increase after the inoculation with mycelium of AO5-m (11%) and Aaw (9.6%). An increment of total phenolics was also observed after the inoculation during 8 days with spores of AO8-s; however, the increase was lower (6.4%). 

Regarding the families of phenolic compounds, simple phenols significantly increased about 38.8% after the inoculation with mycelium of AO5-m. A lower increase was observed after the inoculation with mycelium of Aaw (14.5%) and Anig (17.8%). The inoculation with spores of *Aspergillus oryzae* (AO5-s and AO8-s) did not improve the recovery of simple phenols. The increment was related to the increase of hydroxytyrosol, hydroxytyrosol derivative and hydroxytyrosol ester with lactone, that could be associated to the breakdown of oleuropein (formed by the ester of hydroxytyrosol with elenolic acid) during fermentation. In fact, according to other authors [40,41], *Aspergillus niger* produce β-glycosidase and esterase that are able to hydrolyze the oleuropein. This could be a promising finding because hydroxytyrosol and its derivatives have been considered by EFSA as compounds that contribute to the protection of blood lipids from oxidative stress [42]. Thus, fermentation could be a useful tool to increment the recovery of hydroxytyrosol and its derivatives from olive leaves. 

Elenolic acid derivatives showed a significant increase after all types of fermentation. Inoculation with mycelium and 5-days spores of AO5-s were the fermentations that mostly augment the concentration of elenolic acid derivatives (65.3% and 65.7%, respectively), followed by inoculation with mycelium of Anig and Aaw and inoculation with 8-days spores AO8-s (59.5%, 59.9% and 60.15%, respectively). As exception of elenolic acid glucoside isomer c, the rest of elenolic acid derivatives increased after all types of fermentation. This fact probably indicates that secoiridoids such as oleuropein and ligstroside derivatives suffer a breakdown during fermentation, as stated before during the evaluation of hydroxytyrosol derivatives behavior. This aspect is also important; in fact Salamanca et al. [43] have recently noticed the antiviral activity of this compound. These authors showed as elenolic acid is able to reduce the neuraminidase activity and to prevent the metabolic inactivation of the host cells during the infection experiments, promoting it as anti-influenza compound. 

Secoiridoids presented a significant increase after the olive leaves treatment with mycelium of Aaw and AO8-s (5.4% and 8.4%, respectively); whereas the inoculation with mycelium of *Aspergillus oryzae* AO5-m did not show significant differences compared to control sample and mycelium of Anig and AO5-m showed a significant decrease of secoiridoids extraction (−6.1% and −15.2%, respectively). This is partially justifiable by the enzymatic activity of the *Aspergillus niger* that is a good producer of β-glucosidase [44]; therefore it is plausible to hypothesize that the fermentation with this microorganism promotes the conversion of oleuropein in hydroxytyrosol, thus the content of secoridoids is lower. The compounds that contribute to observe this increment trend are: oleoside, secologanoside, oleuropein isomer a, hydrooleuropein, oleuropein isomer b, oleuropein isomer c, oleuropein isomer d, oleuropein isomer e, oleuropein isomer f, oleuropein isomer g, oleuropein aglycone isomer a. Most of them are isomers of oleuropein and this fact coincides with the reduction of oleuropein glucoside isomers, which maybe can be explain because of the breakdown of the glycosidic bond during fermentation. About minor secoridoids, monohydrated oleacein was detected only in fermented samples and the highest content has been noticed in AO5-m sample. This secoridoid is a hydrolytic derivative of oleuropein and, more precisely, it represents its dialdehydic isomeric form with anticancer properties [45]. Recently, Marx and co-workers [46] noticed as fungi isolated from olive leaves are able to improve the content of oleacein in Arbequina olives. This trend was tentatively justified by these authors by a possible higher production of pectinases and cellulases, which may promote the hydrolysis of the cellular membrane and consequently of the phenolic compounds.

Flavonoids content significantly improved after the inoculation with AO5-s and Aaw (11.5% and 16.8%, respectively). The other strains did not show significant differences with the control sample. Luteolin rutinoside isomer a, luteolin and resinoside isomer b contribute to the high flavonoid levels in both kind of fermentations. However, gallocatechin, apigenin glucoside, chrysoeriol-7-glucoside, quercetin, and resinoside isomer a showed the highest increment after the inoculation with Aaw and luteolin rutinoside isomers b and d presented the highest concentration after the inoculation with AO5-s. The increase of quercetin-glucoside in Aaw sample is consistent with the data reported by Leonard et al. [47]; these authors noticed that the *Aspergillus awamori* is able to release quercetin from rutin thanks to the α-rhamnosidase responsible to the convertion of rutin in quercetin-glucoside, and to the β-glucosidase that catalyze the conversion of quercetin-3-glucoside to quercetin.

Iridoids, represented by allobetonicoside, were only present in the autoclaved sample before fermentation and the other phenolic compounds significantly diminish after fermentation compared to autoclaved sample. This decrease was in the range between 25.4% with Aaw and 39.8% with AO5-m.

**Figure 2:**
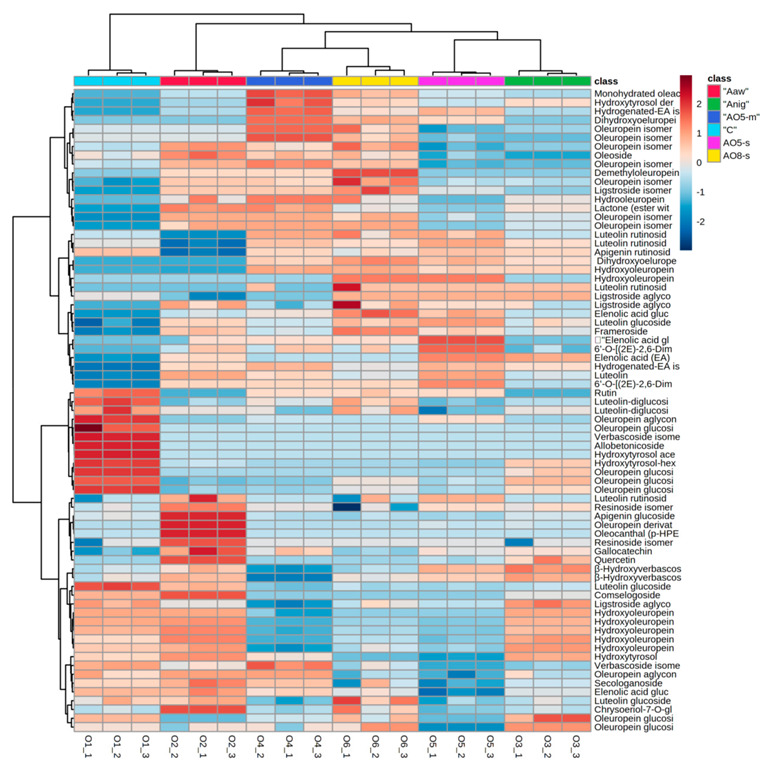
Hierarchical clustering analysis of phenolic compounds across the samples showing the distribution of target phenolic compounds and their concentration.

To better understand the phenolic distribution in the samples, a color-coded two-dimensional heatmap was produced and it is shown in Figure 2. It is composed by two clusters (one sample oriented and the other one is variable-oriented) and was calculated using the Euclidean distance according to the Ward method.

Sample oriented cluster showed a first cluster discriminating control from fermented samples; the second cluster discriminated between Aaw fermented sample and the other ones. Red zones in the map correspond to high amounts of the selected compound. 

Analyzing the results of control samples, it is very clear that the red colored boxes corresponding to oleuropein-derivatives (particularly oleuropein aglycone and glucoside), rutin, luteolin-glucosides depicts the high amounts of these phenolics as compared to other samples. Aaw fermented samples reported a cluster related to the high amounts of apigenin glucoside, quercetin and β-hydroxyverbascoside isomers. Generally, fermented samples showed sub-clusters indicating the higher amounts of elenolic acid derivatives, hydroxytyrosol-derivatives, some oleuropein isomers, and oleoside.

PLS-DA was used to classify the different olive leaf samples using the phenolic composition as predictors. The PLS-DA score plot for the final model is shown in Supplementary Figure S1; it shows as the samples were well clustered according to control and fermented samples as well as a tendency for the different fermentations clustering with a classification according to the microorganism and the form that it was inoculated (spore or mycelium).

It is important to underline that, as previously reported, the increase or decrease of the phenolic compounds is related to the production of specific enzymes by the selected fungi that are able to transform the phenolic structure; however, the increase of the content of phenolic compounds is also due to the action of the lignocellulolytic enzymes produced by the genus Aspergillus [44] that are capable to degrade the cell wall polysaccharides improving the extractability of the phenolic compounds.

**Figure 3:**
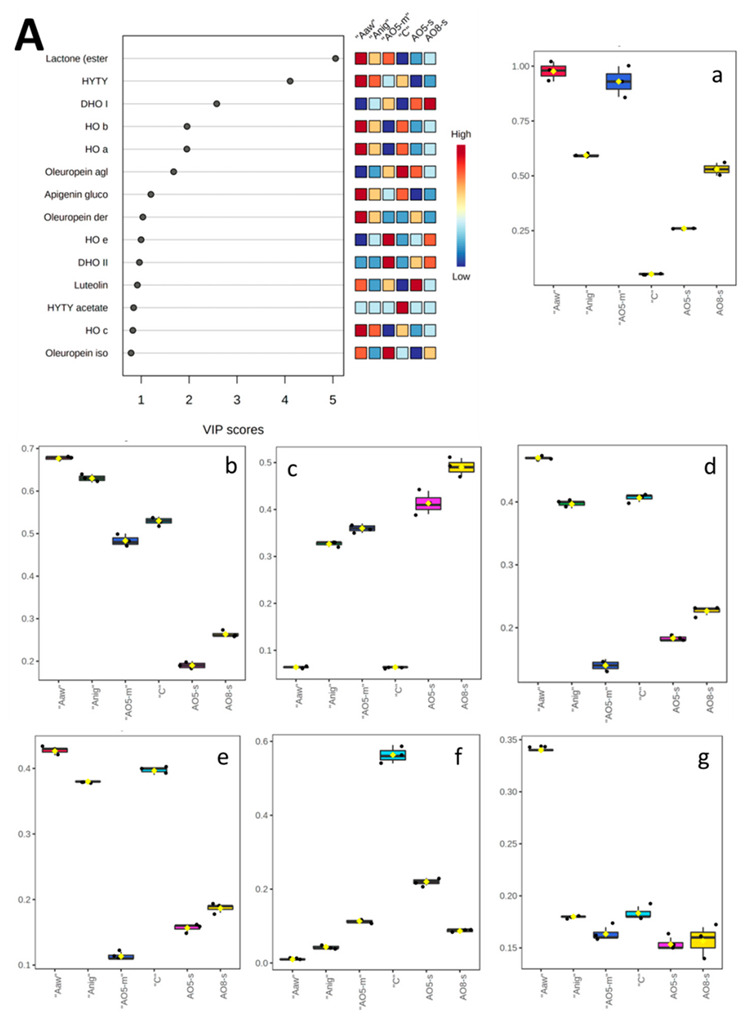
VIP score of PLS-DA analysis (**A**) and box plots (**a**–**g**) reporting the amounts of targeted compounds with VIP score > 1 in the different samples. HYTY, hydroxytyrosol; DHO, dihydroxyoleuropein; HO, hydroxyoleuropein.

Figure 3A reports the results of PLS-DA analysis; the most important phenolic compounds are plotted according to the PLS-DA variable importance in projection (VIP) scores. In this way, the importance of variables to the model was presented and according to other authors [48] threshold was set to VIP > 1. Figure 3a–g reports the concentration (µg/g d.w.) of the selected phenolic compounds with VIP score ranging between 5.06 to 1.20 corresponding to hydroxytyrosol-lactone (a), hydroxytyrosol (b), dihydroxyoleuropein isomer I (c), hydroxyoleuropein isomer I and II (d and e), oleuropein aglicone isomer b (f), and apigenin-glucoside (g). 

On the other hand, the most abundant compound in the olive leaf samples is the hydroxytyrosol lactone ester, this compound has been found in all the fermented samples (Figure 3a) and we suppose that is a metabolite formed during the fermentation; leaves fermented with Aaw and AO5-m showed the highest content. As showed in Figure 3b, hydroxytyrosol was detected in control and fermented samples; however, its content increased after fermentation with Aaw and Anig. Dihydroxyoleuropein isomer I was the third compound in terms of VIP score and its content was higher in samples fermented with spores of *A. oryzae*, lower amounts were also described in the samples fermented with the mycelium of *A. niger* and *A. oryzae*; contrary, this compound was not detected in the sample fermented with *A. awamori*.

About the compounds hydroxyoleuropein isomer I and II, and apigenin glucoside only the fermentation with *A. awamori* was able to improve their content compared to the control. Finally, oleuropein aglycone isomer b is higher in the control and its content decreased during the fermentation confirming the rupture of this molecule to form hydroxytyrosol derivatives [40,41].

### 3.3. Evaluation of Antioxidant Activity in Control and Fermented Extracts

**Figure 4:**
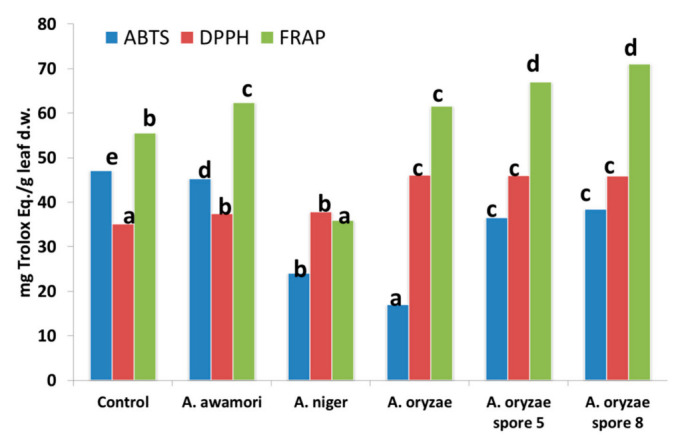
Antioxidant activity (mg Trolox equivalent/g leaf d.w.) of analysed samples. Different letters in the same raw indicate statistical differences (*p* < 0.05).

Figure 4 reported the values of the antioxidant activity of the extracts evaluated by three different assays. As previously reported [32], these assays have different mechanism of action; in fact, ABTS and DPPH mechanism is based on the electron transfer or by radical quenching, besides FRAP mechanism is based on the ability of phenolic compounds to donate an electron.

**Figure 5:**
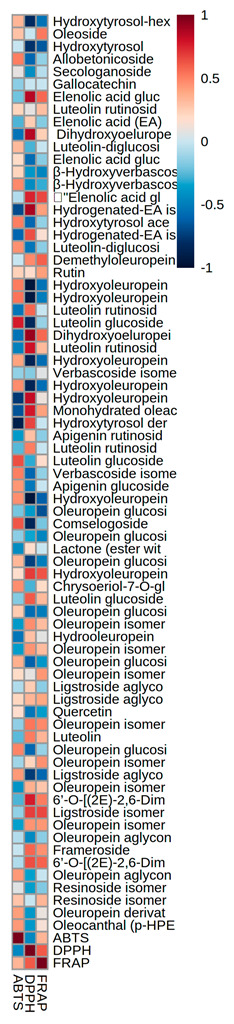
Heatmap visualization of the correlations between phenolic compounds and the antioxidant activity evaluated by ABTS, DPPH and FRAP.

Antioxidant activity evaluated by ABTS assay ranged between 17 and 47.1 mg Trolox equivalent/g leaf d.w. and the control sample showed the highest value. According to the obtained data, all the samples fermented with the *A. oryzae* reported the highest values of antioxidant activity with DPPH radical assay; contrary, control was the lowest one. Briefly, sample fermented by *A. niger* showed the lowest value of antioxidant activity evaluated by FRAP, the samples fermented with the spores of *A. oryzae* showed the highest one. These data confirmed as the different phenolic compounds present different mechanisms of action. Figure 5 reports the Pearson’s correlations between the antioxidant activity and the phenolic composition.

A moderate high correlation was noticed between the luteolin-glucoside and comselogoside, and the ABTS assay. In other words, high correlation was noticed between DPPH and elenolic acid glucoside, dihydroxyoleuropein, hydrogenated elenolic acid, luteoin rutinoside, hydroxyoleuropein, ligstroside and 6′-O-[(2E)-2,6-dimethyl-8-hydroxy-2-octenoyloxy] secologanoside. 

Finally, ligstroside and 6′-O-[(2E)-2,6-dimethyl-8-hydroxy-2-octenoyloxy] secologanoside, hydroxyoleuropein and dihydroxyoleuropein was moderately correlated with FRAP assay.

High correlation was found among the antioxidant assays.

## 4. Conclusions

This study proposed the solid state fermentation as a treatment of olive leaves aiming the increase of hydroxytyrosol and elenolic acid derivatives concentration. 

The use of HPLC-ESI-TOF-MS allowed the tentatively identification of new compounds that, to our knowledge, were not previously described in olive leaves. Most of them were detected only in fermented samples confirming that they are metabolites that have been formed during the fermentation.

Fermentation using mycelium of *Aspergillus awamori*, *Aspergillus niger* and *Aspergillus oryzae* were effective to increase both hydroxytyrosol and elenolic acid derivatives whereas the use of spores of *Aspergillus oryzae* caused a loss of hydroxytyrosoyl derivatives, contrary the content of elenolic derivatives are comparable with the other fermentation treatments and higher than control.

Briefly, the proposed fermentation processes using the mycelium of *Aspergillus awamori*, *Aspergillus niger* and *Aspergillus oryzae* lead to an increase the hydroxytyrosyl and elenolic acid derivatives and could be used at industrial scale to obtain enriched extracts. Further works will be focused on the use of mix of microorganism to evaluate possible synergisms in terms of secoridoid hydrolysis to produce enriched extracts of hydroxytyrosol and elenolic acid derivatives. Moreover, additional researches are needed to better understand the kinetic of formation of fermentation metabolites (i.e., lactone ester with hydroxytyrosol among others) during the process.

## Data Availability

Not applicable.

## Supplementary Materials

The following supporting information can be downloaded at: https://www.mdpi.com/article/10.3390/antiox11091693/s1, Figure S1: PLS-DA analysis; Table S1: Analytical parameters of the method proposed. LOD: Limit of detection, LOQ: Limit of quantification. Table S2: Phenolic compounds tentatively identified in olive leaf extracts by HPLC-ESI-TOF-MS; Table S3: Content of phenolic compounds in olive leaf extracts (mg/g d.w. leaf).
