# Supplementary material for: Solid State Fermentation of Olive Leaves as a Promising Technology to Obtain Hydroxytyrosol and Elenolic Acid Derivatives Enriched Extracts
Source: Antioxidants (Basel). 2022 Aug 29;11(9):1693. doi: 10.3390/antiox11091693 (PMC9496001; doi:10.3390/antiox11091693)

Table S1. Analytical parameters of the method proposed. LOD: Limit of detection, LOQ: Limit of quantification.

**Analyte**	**LOD**	**LOQ**	**Linear range (mg L^{-1})**	**Calibration curves (mg L^{-1})**	**r^2**
Hydroxytyrosol	0.0122	0.0407	LOQ-100	y=76790x + 108991	0.9977
Oleuropein	0.0954	0.3179	LOQ-100	y= 6615.7x + 9603.8	0.9947
Rutin	0.2366	0.7888	LOQ-100	y= 3957.7x + 2144.5	0.9970
Apigenin-7-glucoside	0.0229	0.0764	LOQ-100	y= 40886x + 833.18	0.9988
Luteolin	0.0061	0.0203	LOQ-100	y= 153477x + 4221.2	0.9995

Table S2. Phenolic compounds tentatively identified in olive leaf extracts by HPLC-ESI-TOF-MS

	RT (min)	m/z experimental	m/z calculated	Conf (%)	Error (ppm)	Molecular formula	Compound	C	Aaw	Anig	AO5-m	AO5-s	AO8-s	Ref.
1	1.45	315.1074	315.1080	100	-1.9	C14H20O8	Hydroxytyrosol-hexose	X	X	X	X	X	X	[1,2]
2	1.51	389.1064	389.1084	96.06	-5.1	C16H22O11	Oleoside	X	X	X	X	X	X	[1,2]
3	1.64	153.0546	153.0552	100	-3.9	C8H10O3	Hydroxytyrosol	X	X	X	X	X	X	[1]
4	2.55	505.1552	505.1550	100.00	-1.0	C21H30O14	Allobetonicoside	X	-	-	-	-	-	[3]
5	3.44	389.1076	389.1084	100	-2.1	C16H22O11	Oleoside/secologanoside	X	X	X	X	X	X	[1,2]
6	3.58	305.0682	305.0661	58.17	6.9	C15H14O7	Gallocatechin	X	X	X	X	X	X	Pubchem
7	3.76	403.1233	403.1240	99.95	-1.7	C17H24O11	Elenolic acid glucoside isomer a	X	-	-	-	-	-	[1,2]
8	4.62	403.1235	403.1240	99.95	-1.2	C17H24O11	Elenolic acid glucoside isomer b	X	X	X	X	X	X	[1,2]
9	5.84	593.1494	593.1506	99.92	-2.0	C27H30O15	Luteolin rutinoside isomer a	X	X	X	X	X	X	[1,2]
10	6.03	215.0918	215.0919	100	-0.5	C10H16O5	Elenolic acid (EA) derivative	X	X	X	X	X	X	[4]
11	6.35	403.1230	403.1240	99.95	-2.5	C17H24O11	Elenolic acid glucoside isomer c	X	-	-	-	-	-	[1,2]
12	6.59	571.1658	571.1663	100.00	-0.9	C25H32O15	Dihydroxyoeluropein isomer a	-	-	X	X	X	X	[5]
13	6.72	609.1458	609.1456	99.79	0.3	C27H30O16	Luteolin-diglucoside isomer a	X	X	X	X	X	X	[1,2]
14	6.89	403.1240	403.1240	99.95	-5.7	C17H24O11	Elenolic acid glucoside isomer d	X	X	X	X	X	X	[1,2]
15	7.04	639.1914	639.1925	99.81	-1.7	C29H36O16	β-Hydroxyverbascoside [Campneoside II] isomer a	X	X	X	X	X	X	[6]
16	7.19	639.1918	639.1925	99.99	-1.1	C29H36O16	β-Hydroxyverbascoside [Campneoside II] isomer b	X	X	X	X	X	X	[6]
17	7.52	403.1237	403.1240	99.95	-0.7	C17H24O11	Elenolic acid glucoside isomer e	X	X	X	X	X	X	[1,2]

18	7.63	243.0860	243.0869	100	-3.7	C11H16O6	Hydrogenated-EA isomer a	-	X	X	X	X	X	[4,7]
19	7.89	195.0650	195.0657	100	-3.6	C10H12O4	Hydroxytyrosol acetate	X	-	-	-	-	-	[8, 9]
20	8.08	243.0866	243.0869	100	-1.2	C11H16O6	Hydrogenated-EA isomer b	-	X	X	X	X	X	[4,7]
21	8.16	403.1222	403.1240	99.95	-4.5	C17H24O11	Elenolic acid glucoside isomer f	X	X	X	X	X	X	[1,2]
22	8.21	609.1454	609.1456	100.00	-0.3	C27H30O16	Luteolin-diglucoside isomer b	X	X	X	X	X	X	[1,2]
23	8.35	525.1597	525.1608	99.99	-2.1	C24H30O13	Demethyloleuropein isomer a	X	X	X	X	X	X	[1,2]
24	8.61	609.1437	609.1456	99.99	-3.1	C27H30O16	Rutin	X	-	-	X	X	X	[1,2]
25	8.71	555.1702	555.1714	100.00	-2.2	C25H32O14	Hydroxyoleuropein isomer a	X	X	X	X	X	X	[1]
26	8.79	555.1702	555.1714	100.00	-2.2	C25H32O14	Hydroxyoleuropein isomer b	X	X	X	X	X	X	[1]
27	9.05	593.1497	593.1506	99.99	-1.5	C27H30O15	Luteolin rutinoside isomer b	X	X	X	X	X	X	[1,2]
28	9.06	447.0918	447.0927	100.00	-2.0	C21H20O11	Luteolin glucoside isomer a	X	X	X	X	X	X	[1,2]
29	9.13	571.1663	571.1663	100.00	0.0	C25H32O15	Dihydroxyoeluropein isomer b	-	-	-	X	X	X	[5]
30	9.26	701.2291	701.2293	99.63	-0.3	C31H42O18	Oleuropein glucoside isomer a	X	-	-	-	-	-	[1]
31	9.31	551.1769	551.1765	99.97	0.7	C26H32O13	6-(3-Hydroxy-3-methylbutyl) taxifolin 7-O-β-D-glucoside	-	-	X	X	X	X	Pubchem
32	9.41	525.1605	525.1608	100.00	-0.6	C24H30O13	Demethyloleuropein isomer b	X	-	-	-	-	-	[1,2]
33	9.43	701.2292	701.2293	99.91	-0.1	C31H42O18	Oleuropein glucoside isomer b	X	-	-	-	-	-	[1]
34	9.58	593.1514	593.1506	99.77	1.3	C27H30O15	Luteolin rutinoside isomer c	X	X	X	X	X	X	[1,2]
35	9.62	555.1723	555.1714	80.99	1.6	C25H32O14	Hydroxyoleuropein isomer c	X	X	X	X	X	X	[1]
36	9.74	623.1990	623.1976	99.98	2.2	C29H36O15	Verbascoside isomer a	X	X	X	X	X	X	[1,2]

37	9.82	555.1725	555.1714	99.94	2.0	C25H32O14	Hydroxyoleuropein isomer d	X	X	X	X	X	X	[1]
38	10.09	555.1732	555.1714	99.99	3.2	C25H32O14	Hydroxyoleuropein isomer e	X	-	X	X	X	X	[1]
39	10.24	337.1285	337.1287	100	-0.6	C17H22O7	Monohydrated oleacein	-	X	X	X	X	X	[10]
40	10.35	323.1489	323.1495	100	-1.9	C17H24O6	Hydroxytyrosol derivative	-	X	X	X	X	X	[11]
41	10.42	577.1575	577.1557	99.95	3.1	C27H30O14	Apigenin rutinoside	X	X	X	X	X	X	[1,2]
42	10.44	593.1505	593.1506	99.97	-0.2	C27H30O15	Luteolin rutinoside isomer d	X	X	X	X	X	X	[1,2]
43	10.49	447.0915	447.0927	100.00	-2.7	C21H20O11	Luteolin glucoside isomer b	X	X	X	X	X	X	[1,2]
44	10.55	623.1989	623.1976	99.90	2.1	C29H36O15	Verbascoside isomer b	X	-	-	X	-	-	[1,2]
45	10.64	431.0976	431.0978	100	-0.5	C21H20O10	Apigenin glucoside	X	X	X	X	X	X	[1]
46	10.72	701.2292	701.2293	100.00	-0.1	C31H42O18	Oleuropein glucoside isomer c	X	-	-	-	-	-	[1]
47	10.84	555.1718	555.1714	99.96	0.7	C25H32O14	Hydroxyoleuropein isomer f	X	X	X	X	X	X	[1]
48	10.86	701.2289	701.2293	99.98	-0.6	C31H42O18	Oleuropein glucoside isomer d	X	X	X	X	X	X	[1]
49	10.88	535.1810	535.1816	99.79	-1.1	C25H28O13	Comselogoside	X	X	X	-	-	-	[4]
50	10.97	623.1968	623.1976	99.89	-1.3	C29H36O15	Verbascoside isomer c	-	X	-	-	-	-	[1,2]
51	11.09	701.2301	701.2293	99.62	1.1	C31H42O18	Oleuropein glucoside isomer e	X	X	X	X	X	X	[1]
52	11.35	321.1332	321.1338	100	-1.9	C17H22O6	Lactone (ester with hydroxytyrosol)	-	X	X	X	X	X	[11]
53	11.44	701.2296	701.2293	99.99	0.4	C31H42O18	Oleuropein glucoside isomer f	X	-	X	X	X	X	[1]
54	11.51	461.1085	461.1084	100	0.2	C22H22O11	Hydroxyoleuropein isomer g	-	-	-	X	X	X	[1]
55	11.58	555.1704	555.1714	99.89	-1.8	C25H32O14	Chrysoeriol-7-Oglucoside	X	X	X	X	X	X	[1,2]
56	11.63	447.0927	447.0927	99.97	0.0	C21H20O11	Luteolin glucoside isomer c	X	X	X	X	X	X	[1,2]
57	11.84	701.2300	701.2293	99.59	1.0	C31H42O18	Oleuropein glucoside isomer g	X	X	X	X	X	X	[1]

58	12.35	539.1770	539.1765	91.85	0.9	C25H32O13	Oleuropein isomer a	X	X	X	X	X	X	[1,2]
59	12.43	541.1932	541.1921	99.99	2.0	C25H34O13	Hydro-oleuropein	X	X	X	X	X	X	[4]
60	12.56	539.1771	539.1765	98.15	1.1	C25H32O13	Oleuropein isomer b	X	X	X	X	X	X	[1,2]
61	12.59	569.1874	569.1870	100.00	0.7	C26H34O14	2''-Methoxyoleuropein isomer a	X	-	-	-	X	-	[2]
62	12.76	569.1872	569.1870	100.00	0.4	C26H34O14	2''-Methoxyoleuropein isomer b	X	X	X	X	X	X	[2]
63	12.90	701.2292	701.2293	99.72	-0.1	C31H42O18	Oleuropein glucoside isomer h	X	X	X	X	X	X	[1]
64	13.01	539.1777	539.1765	100.00	2.2	C25H32O13	Oleuropein isomer c	X	X	X	X	X	X	[1,2]
65	13.63	537.1608	537.1608	99.28	0.0	C25H30O13	Ligstroside aglycone glucuronide isomer a	X	X	X	X	X	X	[12]
66	13.90	537.1611	537.1608	99.92	0.6	C25H30O13	Ligstroside aglycone glucuronide isomer b	X	X	X	X	X	X	[12]
67	14.15	301.0341	301.0348	100	-2.3	C15H10O7	Quercetin	X	X	X	X	X	X	[13]
68	14.21	539.1761	539.1765	100.00	-0.7	C25H32O13	Oleuropein isomer d	X	X	X	X	X	X	[1,2]
69	14.32	285.0399	285.0399	100	0.0	C15H10O6	Luteolin	X	X	X	X	X	X	[1,2]
70	14.47	701.2275	701.2293	99.98	-2.6	C31H42O18	Oleuropein glucoside isomer i	X	X	X	X	-	X	[1,2]
71	14.66	701.2293	701.2293	100.00	0.0	C31H42O18	Oleuropein glucoside isomer j	X	X	X	X	-	-	[1,2]
72	14.86	539.1755	539.1765	100.00	-1.9	C25H32O13	Oleuropein isomer e	X	X	X	X	X	X	[1]
73	15.14	537.1607	537.1608	98.40	-0.2	C25H30O13	Ligstroside aglycone glucuronide isomer c	X	X	X	X	X	X	[12]
74	15.35	539.1761	539.1765	100.00	-0.7	C25H32O13	Oleuropein isomer f	X	X	X	X	X	X	[1]
75	15.47	583.2034	583.2027	100.00	1.2	C27H36O14	Lucidumoside C isomer a	-	-	-	-	-	-	[5]
76	15.59	557.2231	557.2234	99.97	-0.5	C26H38O13	6′-O-[(2E)-2.6-Dimethyl-8-hydroxy-2-octenoyloxy] secologanoside isomer a	X	X	X	X	X	X	[8]
77	15.63	583.2036	583.2027	99.98	1.5	C27H36O14	Lucidumoside C isomer b	X	-	-	-	-	-	[5]
78	15.77	523.1822	523.1816	99.99	1.1	C25H32O12	Ligstroside	X	X	X	X	X	X	[1,2]

79	15.85	539.1777	539.1765	99.25	2.2	$C_{25}H_{32}O_{13}$	Oleuropein isomer g	X	X	X	X	X	X	[1,2]
80	16.04	377.1232	377.1236	100	-1.1	$C_{19}H_{22}O_{8}$	Oleuropein aglycone	X	X	X	X	X	X	[13]
81	16.13	601.2128	601.2132	99.99	-0.7	$C_{27}H_{38}O_{15}$	Frameroside/2"-epi-frameroside	X	X	X	X	X	X	[14]
82	16.20	553.1922	553.1921	99.73	0.2	$C_{26}H_{34}O_{13}$	Oleuroside methyl ether isomer a	X	X	-	-	-	-	[1]
83	16.37	623.1405	623.1401	100.00	0.6	$C_{31}H_{28}O_{14}$	Luteolin derivative	X	X	-	X	-	X	[4]
84	16.92	583.2041	583.2027	99.99	2.4	$C_{27}H_{36}O_{14}$	Lucidumoside C isomer c	X	-	-	-	-	-	[5]
85	17.56	553.1924	553.1921	100.00	0.5	$C_{26}H_{34}O_{13}$	Oleuroside methyl ether isomer b	X	X	X	X	X	X	[1]
86	17.64	557.2236	557.2234	99.99	0.4	$C_{26}H_{38}O_{13}$	6′-O-[(2E)-2.6-Dimethyl-8-hydroxy-2-octenoyloxy] secologanoside isomer b	X	X	X	X	X	X	[8]
87	17.98	299.0553	299.0556	100	-1.0	$C_{16}H_{12}O_{6}$	Diosmetin	X	X	X	X	X	X	[4]
88	18.73	539.1765	539.1765	100.00	0.0	$C_{25}H_{32}O_{13}$	Oleuropein isomer h	X	-	-	-	-	-	[1,2]
89	18.79	539.1776	539.1765	99.30	2.0	$C_{25}H_{32}O_{13}$	Oleuropein isomer i	X	-	-	-	-	-	[1,2]
90	18.84	539.1780	539.1765	99.98	2.8	$C_{25}H_{32}O_{13}$	Oleuropein isomer j	X	-	-	-	-	-	[1,2]
91	18.89	377.1228	377.1236	100	-2.1	$C_{19}H_{22}O_{8}$	Oleuropein aglycone	X	X	X	X	X	X	[13]
92	18.99	539.1769	539.1765	100.00	0.7	$C_{25}H_{32}O_{13}$	Oleuropein isomer k	X	-	-	-	-	-	[1,2]
93	19.12	613.1921	613.1921	99.74	0.0	$C_{31}H_{34}O_{13}$	Resinoside isomer a	X	X	X	X	X	X	[1]
94	19.40	613.1924	613.1921	99.98	0.5	$C_{31}H_{34}O_{13}$	Resinoside isomer b	X	X	X	X	X	X	[1]
95	21.57	707.2899	707.2915	99.94	-2.3	$C_{35}H_{48}O_{15}$	Oleuropein derivative	X	X	X	X	X	X	[5]
96	22.69	303.1226	303.1232	100	-2.0	$C_{17}H_{20}O_{5}$	Oleocanthal (p-HPEA-EDA)	-	X	X	X	X	X	[4]

1. Martín-García B et al. DOI: 10.1016/j.indcrop.2020.112741
2. Talhaoui N et al. DOI: 10.1016/j.lwt.2014.03.014.
3. Cádiz-Gurrea ML et al. DOI: 10.3390/antiox10020245
4. Abbattista R et al. DOI: 10.3390/foods10061236

5. Contreras MM et al. DOI: 10.3390/foods10010111
6. Cardinali A. et al. DOI: 10.1021/jf204001p
7. Silva S. et al. DOI: 10.1002/mnfr.201700065
8. Quirantes-Piné R et al. DOI: 10.1002/pca.2401
9. Kritikou E et al. DOI: 10.3390/molecules25214889
10. Ventura G et al. DOI: 10.1002/rcm.8514
11. Garcia-Aloy M et al. DOI: 10.3390/metabo11100692
12. Dias MC et al. DOI: 10.1016/j.phytochem.2019.112199.
13. Olmo-García L et al. DOI:10.3390/molecules23102524
14. Sidda JD et al. DOI: 10.1038/s41598-020-76140-z

Table S3. Content of phenolic compounds in olive leaf extracts (mg/g d.w. leaf)

	**Phenolic compound**	**Control**		**Aaw**		**Anig**		**AO5-m**		**AO5-s**		**AO8-s**	
		Mean	SD	Mean	SD	Mean	SD	Mean	SD	Mean	SD	Mean	SD
**1**	Hydroxytyrosol-hexose	0.46	0.01	0.09	0.004	0.23	0.003	0.04	0.002	0.002	0.00003	0.04	0.001
**2**	Oleoside	0.22	0.01	0.25	0.005	0.18	0.001	0.23	0.001	0.19	0.01	0.23	0.01
**3**	Hydroxytyrosol	0.53	0.01	0.68	0.004	0.63	0.02	0.48	0.02	0.19	0.01	0.26	0.01
**4**	Allobetonicoside	0.09	0.01										
**5**	Secologanoside	0.37	0.0001	0.41	0.02	0.34	0.001	0.38	0.001	0.29	0.02	0.36	0.04
**6**	Gallocatechin	0.56	0.02	0.68	0.03	0.62	0.002	0.63	0.02	0.61	0.01	0.59	0.01
**7**	Elenolic acid glucoside isomer a												
**8**	Elenolic acid glucoside isomer b	0.08	0.004	0.13	0.01	0.13	0.001	0.15	0.003	0.18	0.01	0.21	0.01
**9**	Luteolin rutinoside isomer a	0.09	0.01	0.10	0.004	0.09	0.001	0.09	0.01	0.10	0.001	0.09	0.003
**10**	Elenolic acid (EA) derivative	0.15	0.01	0.30	0.005	0.34	0.01	0.24	0.01	0.37	0.003	0.24	0.0002
**12**	Dihydroxyoeluropein isomer I					0.33	0.01	0.36	0.01	0.41	0.04	0.46	0.06
**13**	Luteolin-diglucoside isomer a	0.17	0.01	0.09	0.01	0.10	0.002	0.11	0.01	0.08	0.01	0.14	0.01
**14**	Elenolic acid glucoside isomer c	0.19	0.01	0.20	0.01	0.16	0.003	0.18	0.002	0.10	0.01	0.16	0.004
**15**	β-Hydroxyverbascoside [Campneoside II] isomer I	0.24	0.01	0.29	0.03	0.33	0.01	0.18	0.004	0.30	0.01	0.24	0.002
**16**	β-Hydroxyverbascoside [Campneoside II] isomer II	0.26	0.02	0.30	0.01	0.31	0.01	0.17	0.002	0.29	0.001	0.26	0.002
**17**	Elenolic acid glucoside isomer d			0.01	0.004	0.0029	0.0003	0.01	0.001	0.02	0.0002	0.01	0.0004
**18**	Hydrogenated-EA isomer a			0.08	0.003	0.08	0.003	0.25	0.002	0.18	0.02	0.14	0.0002
**19**	Hydroxytyrosol acetate	0.85	0.01										
**20**	Hydrogenated-EA isomer b			0.33	0.01	0.32	0.003	0.39	0.01	0.38	0.02	0.30	0.005
**22**	Luteolin-diglucoside isomer b	0.08	0.01	0.07	0.002	0.07	0.001	0.06	0.01	0.06	0.004	0.06	0.01
**23**	Demethyloleuropein isomer a			0.01	0.00004	0.0035	0.0004	0.01	0.0004			0.02	0.0003
**24**	Rutin	0.10	0.002	0.002	0.001			0.06	0.001	0.06	0.002	0.07	0.001
**25**	Hydroxyoleuropein isomer a	0.40	0.01	0.43	0.01	0.38	0.004	0.11	0.01	0.16	0.01	0.19	0.001

**26**	Hydroxyoleuropein isomer b	0.41	0.01	0.47	0.004	0.40	0.01	0.14	0.01	0.18	0.01	0.22	0.001
**27**	Luteolin rutinoside isomer b	0.08	0.002	0.02	0.004	0.09	0.0002	0.10	0.003	0.11	0.004	0.09	0.01
**28**	Luteolin glucoside isomer a	0.61	0.01	0.55	0.01	0.49	0.01	0.45	0.01	0.47	0.01	0.43	0.05
**29**	Dihydroxyoeluropein isomer II							0.21	0.004	0.12	0.002	0.16	0.005
**33**	Oleuropein glucoside isomer a												
**34**	Luteolin rutinoside isomer c	0.10	0.001	0.07	0.002	0.10	0.001	0.13	0.002	0.13	0.003	0.11	0.0004
**35**	Hydroxyoleuropein isomer c	0.12	0.002	0.17	0.005	0.16	0.004	0.04	0.003	0.06	0.01	0.08	0.001
**36**	Verbascoside isomer a	0.88	0.003	0.81	0.01	0.75	0.01	0.91	0.03	0.71	0.002	0.78	0.04
**37**	Hydroxyoleuropein isomer d	0.12	0.001	0.14	0.005	0.14	0.001	0.05	0.004	0.07	0.01	0.09	0.004
**38**	Hydroxyoleuropein isomer e	0.01	0.002			0.16	0.002	0.21	0.01	0.16	0.01	0.21	0.002
**39**	Monohydrated oleacein			0.09	0.001	0.08	0.001	0.25	0.01	0.10	0.002	0.17	0.002
**40**	Hydroxytyrosol derivative			0.42	0.01	0.83	0.02	1.51	0.22	0.54	0.01	0.93	0.000
**41**	Apigenin rutinoside	0.17	0.002	0.06	0.01	0.16	0.0009	0.16	0.003	0.17	0.003	0.15	0.02
**42**	Luteolin rutinoside isomer d	0.02	0.0001	0.02	0.0003	0.03	0.0003	0.02	0.002	0.03	0.002	0.03	0.002
**43**	Luteolin glucoside isomer b	0.34	0.02	0.35	0.01	0.32	0.01	0.31	0.01	0.31	0.01	0.33	0.01
**44**	Verbascoside isomer b	0.38	0.003										
**45**	Apigenin glucoside	0.18	0.01	0.34	0.004	0.18	0.0016	0.16	0.01	0.15	0.01	0.16	0.02
**46**	Oleuropein glucoside isomer b												
**47**	Hydroxyoleuropein isomer f	0.03	0.004	0.03	0.002	0.03	0.00004	0.005	0.001	0.01	0.002	0.007	0.0001
**48**	Oleuropein glucoside isomer c	0.02	0.001			0.03	0.001	0.01	0.003			0.02	0.0001
**49**	Comselogoside	0.02	0.0001	0.03	0.002	0.01	0.0003						
**50**	Verbascoside isomer c												
**51**	Oleuropein glucoside isomer d	0.02	0.002	0.02	0.005	0.03	0.0002	0.02	0.004	0.001	0.002	0.03	0.00001
**52**	Lactone (ester with hydroxytyrosol)			0.98	0.06	0.59	0.0007	0.93	0.10	0.26	0.01	0.55	0.01
**53**	Oleuropein glucoside isomer e	0.06	0.0005			0.03	0.0026					0.002	0.00003
**54**	Hydroxyoleuropein isomer g									0.01	0.003	0.01	0.0001
**55**	Chrysoeriol-7-Oglucoside	0.29	0.01	0.36	0.002	0.29	0.003	0.29	0.01	0.28	0.01	0.29	0.02
**56**	Luteolin glucoside isomer c	0.20	0.02	0.27	0.002	0.26	0.01	0.25	0.004	0.29	0.002	0.28	0.01

**57**	Oleuropein glucoside isomer f	0.16	0.002	0.02	0.001	0.11	0.004	0.02	0.003	0.04	0.01	0.07	0.0005
**58**	Oleuropein isomer a			0.05	0.001	0.03	0.001	0.05	0.003	0.02	0.003	0.05	0.0001
**59**	Hydrooleuropein	0.0009	0.00001	0.01	0.003	0.01	0.002	0.02	0.001			0.01	0.0001
**60**	Oleuropein isomer b	0.03	0.002	0.07	0.005	0.05	0.001	0.07	0.003	0.04	0.004	0.07	0.001
**61**	2''-Methoxyoleuropein isomer a												
**62**	2''-Methoxyoleuropein isomer b												
**63**	Oleuropein glucoside isomer g	0.59	0.01	0.14	0.06	0.33	0.01	0.16	0.01	0.18	0.03	0.27	0.04
**64**	Oleuropein isomer c	2.06	0.03	2.52	0.03	1.94	0.02	2.27	0.08	1.80	0.12	2.38	0.06
**65**	Ligstroside aglycone glucuronide isomer a	0.07	0.0005	0.05	0.002	0.08	0.002	0.06	0.001	0.08	0.000	0.08	0.0002
**66**	Ligstroside aglycone glucuronide isomer b	0.13	0.003	0.16	0.01	0.14	0.01	0.14	0.01	0.15	0.002	0.15	0.0004
**67**	Quercetin			0.07	0.001	0.05	0.01			0.01	0.004		
**68**	Oleuropein isomer d	0.22	0.01	0.31	0.0004	0.26	0.01	0.30	0.01	0.26	0.01	0.33	0.01
**69**	Luteolin	0.04	0.03	0.55	0.01	0.33	0.02	0.45	0.02	0.57	0.01	0.36	0.004
**70**	Oleuropein glucoside isomer h	0.02	0.002										
**71**	Oleuropein glucoside isomer i	0.002	0.001			0.0007	0.0002						
**72**	Oleuropein isomer e	1.09	0.02	1.34	0.01	0.94	0.01	1.43	0.03	0.91	0.05	1.22	0.13
**73**	Ligstroside aglycone glucuronide isomer c	0.20	0.01	0.17	0.001	0.20	0.003	0.13	0.003	0.16	0.003	0.18	0.002
**74**	Oleuropein isomer f	0.10	0.0003	0.10	0.001	0.09	0.004	0.14	0.001	0.08	0.005	0.11	0.0003
**75**	Lucidumoside C isomer a												
**76**	6′-O-[(2E)-2,6-Dimethyl-8-hydroxy-2-octenoyloxy] secologanoside isomer I	0.003	0.001	0.03	0.001	0.02	0.0009	0.03	0.004	0.04	0.002	0.03	0.0002
**77**	Lucidumoside C isomer b												
**78**	Ligstroside isomer b	0.01	0.0002	0.04	0.003	0.02	0.0008	0.04	0.0002	0.03	0.004	0.06	0.003
**79**	Oleuropein isomer g	0.07	0.0007	0.07	0.0002	0.05	0.0004	0.11	0.0004	0.05	0.005	0.08	0.001
**80**	Oleuropein aglycone isomer a	0.05	0.003	0.06	0.0001	0.04	0.002	0.06	0.0007	0.03	0.004	0.04	0.000

**81**	Frameroside	0.19	0.01	0.32	0.01	0.28	0.01	0.27	0.01	0.31	0.02	0.36	0.003
**84**	Lucidumoside C isomer c												
**86**	6′-O-[(2E)-2,6-Dimethyl-8-hydroxy-2-octenoyloxy] secologanoside isomer II	0.001	0.002	0.015	0.001	0.004	0.003	0.02	0.004	0.03	0.001	0.02	0.00001
**88**	Oleuropein isomer h												
**89**	Oleuropein isomer i												
**90**	Oleuropein isomer j												
**91**	Oleuropein aglycone isomer b	0.56	0.03	0.01	0.001	0.04	0.01	0.11	0.01	0.22	0.02	0.10	0.002
**92**	Oleuropein isomer k												
**93**	Resinoside isomer a	0.08	0.01	0.13	0.004	0.095	0.001	0.09	0.001	0.10	0.0001	0.09	0.002
**94**	Resinoside isomer b	0.03	0.004	0.04	0.001	0.025	0.002	0.03	0.001	0.03	0.001	0.03	0.001
**95**	Oleuropein derivative			0.17	0.002	0.015	0.002			0.01	0.003		
**96**	Oleocanthal (p-HPEA-EDA)			0.03	0.004								
	**Simple phenols**	1.84	0.01	2.16	0.06	2.24	0.04	2.96	0.34	0.99	0.01	1.79	0.00
	**Elenolic acid derivatives**	0.42	0.02	1.05	0.04	1.04	0.01	1.21	0.001	1.22	0.01	1.06	0.01
	**Flavonoids**	3.15	0.14	3.79	0.06	3.29	0.07	3.40	0.23	3.56	0.02	3.30	0.13
	**Secoiridoids**	7.37	0.11	7.71	0.17	6.95	0.02	7.46	0.21	6.20	0.37	7.77	0.44
	**Iridoids**	0.09	0.01										
	**Other phenolic compounds**	1.76	0.03	1.40	0.03	1.39	0.01	1.26	0.04	1.31	0.01	1.28	0.04
	**Total phenols**	14.64	0.07	16.12	0.24	14.91	0.03	16.30	0.59	13.29	0.38	15.20	0.61

Missing values are < LOQ

Figure S1. PLS-DA analysis

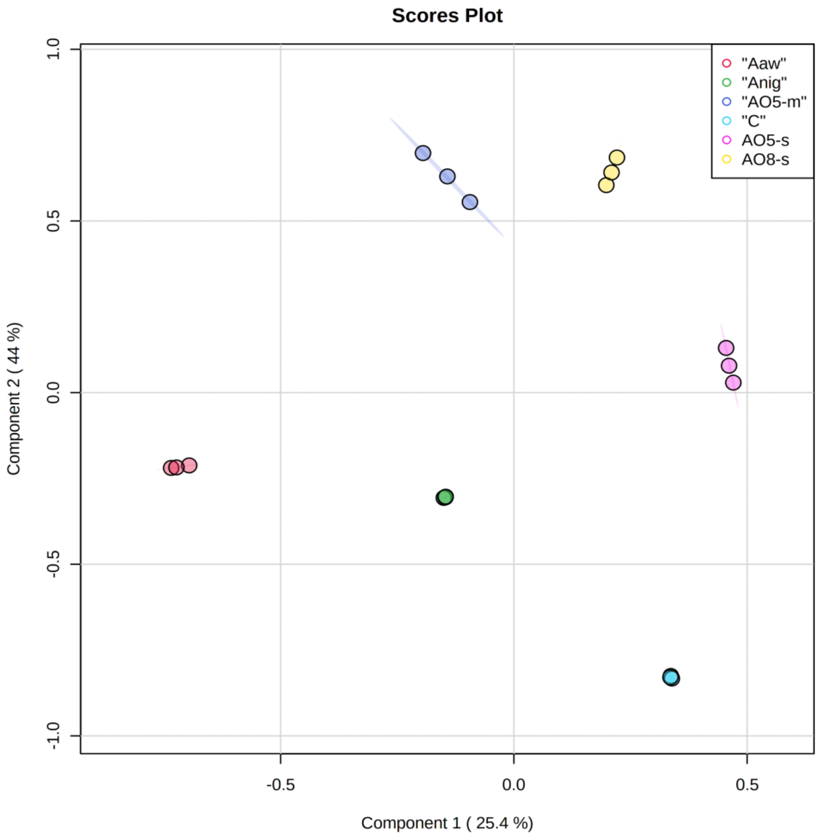

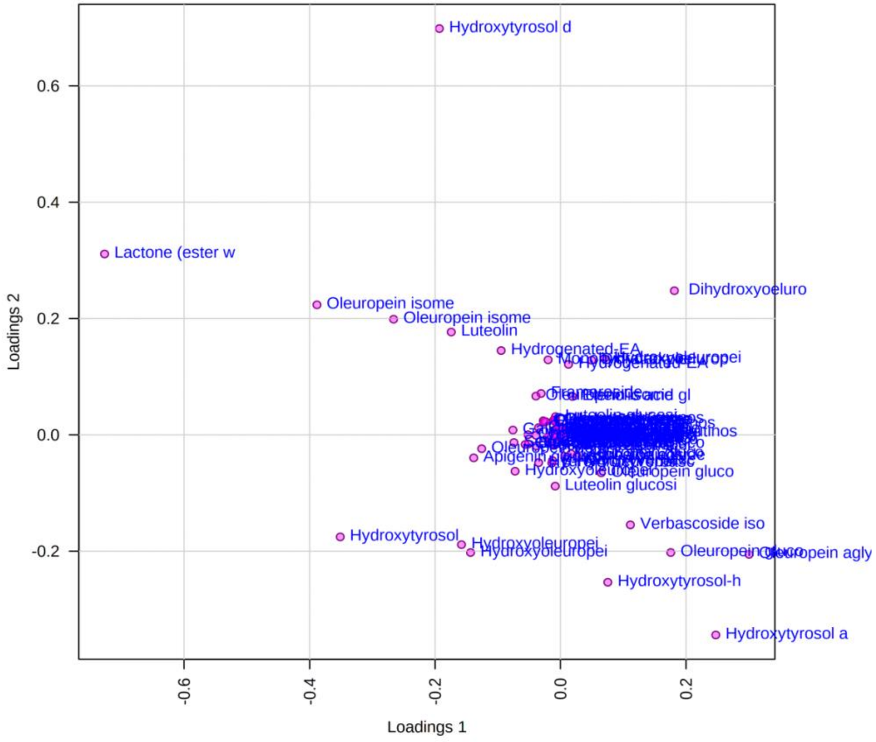

Supplement: Supplementary file 1 [file antioxidants-11-01693-s001.zip › antioxidants-1862131-supplementary.pdf]
